# Development and Characterization of a Cold Cream with Antioxidant Properties from *Bougainvillea* Extract

**DOI:** 10.3390/ph19060932

**Published:** 2026-06-12

**Authors:** Yahya Alhamhoom, Umme Hani, Nagashubha Bobbarjang, Md Abdur Rashid, Srilekha Surapareddy, Kiran Sai Maccha, Uma Maheshwar Rao Vattikuti, Fahad AlQahtani

**Affiliations:** 1 Department of Pharmaceutics, College of Pharmacy, King Khalid University, Abha 62223, Saudi Arabia; ysalhamhoom@kku.edu.sa (Y.A.); uahmed@kku.edu.sa (U.H.); mdrashid@kku.edu.sa (M.A.R.); 2 Department of Pharmaceutics, Raghavendra Institute of Pharmaceutical Education and Research Campus, Ananthapuramu 515721, Andhra Pradesh, India; sirisurapureddy99@gmail.com (S.S.); kiransaimks15502@gmail.com (K.S.M.); 3 Department of Pharmacognosy, CMR College of Pharmacy, Kandlakoya, Medchal Road, Hyderabad 501401, Telangana, India; raovattikuti@gmail.com; 4 Department of Pharmaceutical Sciences, College of Pharmacy, Umm Al-Qura University, Makkah 24381, Saudi Arabia

**Keywords:** *Bougainvillea glabra*, cold cream, HPTLC, DPPH, skin irritation, stability

## Abstract

**Background:** Oxidative stress contributes significantly to premature skin aging and inflammatory dermatological conditions. While plant-derived antioxidants have demonstrated considerable promise in topical applications, *Bougainvillea glabra* Choisy remains underexplored in standardized pharmaceutical dosage form development despite its documented phytochemical richness. **Objective:** This study aimed to develop, standardize, and characterize topical cold cream formulations incorporating *B. glabra* ethanolic leaf extract, with HPTLC-based quantification of marker compounds, validated antioxidant assessment, and preliminary dermal safety evaluation. **Methods:** The ethanolic leaf extract was prepared by maceration and characterized by preliminary phytochemical screening and HPTLC fingerprinting with quantitative densitometric analysis of quercetin and pinitol. Three cold cream formulations were developed at 10% (F1), 20% (F2), and 30% (*w*/*w*) (F3) extract loading. Formulations were evaluated for organoleptic properties, pH, homogeneity, spreadability, and viscosity. Antioxidant activity was assessed using a validated methanol extraction procedure followed by DPPH radical scavenging and potassium permanganate reduction assays. Ex vivo skin permeation was evaluated using Franz diffusion cells with freshly excised goat skin. Accelerated stability was conducted at 40 ± 2 °C/75 ± 5% RH for 90 days with HPTLC-based marker retention monitoring. Primary dermal safety was assessed in Wistar albino rats (*n* = 6) following OECD Test Guideline 404. **Results:** Quantitative HPTLC confirmed quercetin (4.82 ± 0.14 mg/g dry extract) and pinitol (2.31 ± 0.09 mg/g) as marker compounds, with linearly increasing content across F1–F3. All formulations demonstrated acceptable physicochemical properties (pH 5.7–5.9, viscosity 440,000–460,000 cP, spreadability 11.8 ± 0.3 cm·g/s). F3 exhibited the highest DPPH scavenging activity (56.68 ± 1.05%) with IC_50_ of 1.3 ± 0.1% *w*/*v*, demonstrating a 3.2-fold improvement over F1. Extraction recovery from the cream matrix was 96.4–97.1%, validating the antioxidant data. Ex vivo quercetin permeation through goat skin reached 51.3 ± 2.8 μg/cm^2^ at 24 h for F3, following Higuchi diffusion kinetics (R^2^ > 0.99). No dermal irritation was observed (Primary Irritation Index = 0). Accelerated stability confirmed ≥98.3% retention of both marker compounds and antioxidant activity after 90 days. **Conclusions:** *B. glabra* leaf extract was successfully incorporated into a physicochemically stable, non-irritating cold cream with demonstrated dose-dependent antioxidant efficacy and cutaneous delivery capability. The study establishes preliminary dermal safety and in vitro antioxidant efficacy warranting further controlled clinical evaluation.

## 1. Introduction

Oxidative stress, resulting from an imbalance between the production of free radicals and the body’s antioxidant defenses, is a major contributing factor to skin aging and various dermatological conditions [1,2]. Free radicals, highly reactive molecules with unpaired electrons, can damage cellular components like DNA, proteins, and lipids, leading to premature aging, wrinkles, inflammation, and even skin cancer [3]. Antioxidants, which effectively neutralize these free radicals, are therefore crucial in protecting the skin from such damage [4]. The cosmetic and pharmaceutical industries are increasingly turning to natural sources of antioxidants, particularly plant extracts, due to their perceived safety and efficacy [5,6,7]. Among these plant-derived antioxidants, compounds such as curcumin have been extensively investigated for topical applications and have demonstrated proof-of-concept for the successful incorporation of herbal actives into stable dermatological formulations [8]. These precedents highlight the broader scientific opportunity to develop standardized topical products from other botanicals with established antioxidant phytochemistry, yet this potential remains largely unrealized for many plant genera with comparable or superior bioactive profiles.

While compounds such as curcumin have demonstrated proof-of-concept for herbal topical antioxidant formulations, the *Bougainvillea* genus remains scientifically underexplored in standardized dosage form development despite its documented phytochemical richness [9]. *Bougainvillea*, a genus of vibrant flowering plants with a long history of use in traditional medicine systems, has been reported to contain a diverse array of bioactive compounds including flavonoids, betacyanins, and phenolic acids [3]—all well-recognized antioxidant classes that position it as a strong candidate for topical formulation development. These compounds have demonstrated potent antioxidant properties in various in vitro studies [2]. This research explores the potential of incorporating *Bougainvillea* extract into a cold cream formulation, aiming to deliver its antioxidant benefits topically. Cold creams, widely recognized for their moisturizing and cleansing properties, serve as an ideal vehicle for delivering active ingredients to the skin [1]. The formulation and evaluation of herbal creams, such as those containing *Curcuma longa*, have been explored in other research [10], highlighting the suitability of this dosage form for natural extracts [11]. By combining the established benefits of cold cream with the potential antioxidant power of *Bougainvillea* extract, this study seeks to develop a novel topical formulation for skin health.

The novelty of this study lies in its integrated approach to herbal formulation development. While the antioxidant properties of *Bougainvillea* are known, the scientific literature lacks a standardized topical prototype that links chemical fingerprinting with formulation stability and safety [12]. This research is among the first to utilize HPTLC-based quantification of Quercetin and Pinitol as a quality control tool for a *Bougainvillea glabra* cold cream, providing a validated pathway for transforming traditional knowledge into a modern, standardized dermatological product.

Despite the therapeutic potential of *Bougainvillea glabra*, its utilization in standardized topical dosage forms remains under-explored. Most existing studies focus on crude extracts without establishing chemical markers or formulation stability.

Therefore, the primary objective of this study was to bridge this gap by:

Establishing a chemical profile using HPTLC for standardization.

Developing stable cold cream formulations at varying concentrations (10%, 20%, and 30% *w*/*w*).

Evaluating the antioxidant efficacy and dermal safety of the formulated cream [4,13,14]. This research provides a comprehensive framework for transforming a traditional plant extract into a standardized topical formulation with demonstrated preliminary dermal safety and in vitro antioxidant efficacy. Controlled clinical evaluation in populations affected by oxidative stress-related dermatological conditions will be necessary to establish therapeutic efficacy in future studies.

## 2. Results

The formulated *Bougainvillea* extract cold cream was subjected to a series of evaluations, and the results are presented below:

### 2.1. Chemical Standardization by HPTLC

**Figure 1:**
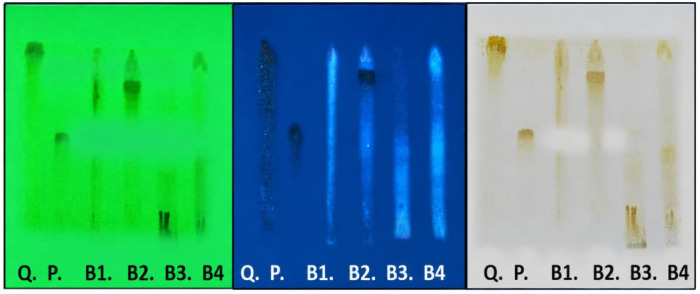
HPTLC fingerprint profile of ethanolic *Bougainvillea glabra* leaf extract and reference standards under three detection modes. Lanes: Q = quercetin reference standard; P = pinitol reference standard; B1–B4 = *B. glabra* ethanolic extract at increasing application volumes (2, 4, 6, and 8 μL). **Left panel**: UV 254 nm—dark quenching bands on fluorescent green background; **centre panel**: UV 366 nm—fluorescent bands showing characteristic quercetin fluorescence at R_f_ 0.52; **right panel**: visible light after derivatization with anisaldehyde-sulfuric acid reagent—brown bands at R_f_ 0.52 (quercetin) and R_f_ 0.35 (pinitol). Mobile phase: toluene:ethyl acetate:formic acid (5:4:1 *v*/*v*/*v*); stationary phase: Silica Gel 60F_254_ HPTLC plates.

The HPTLC fingerprinting of the methanolic extract of *Bougainvillea glabra* revealed a complex profile of phytochemicals. The presence of the marker compounds Quercetin and Pinitol was confirmed by comparing the R_f_ values and the characteristic colors of the bands with their respective reference standards (Figure 1 and Table 1).

#### 2.1.1. Identification of Quercetin

Under UV 366 nm post-derivatization, the extract (B1–B4) showed a bright yellow-fluorescent band at an R_f_ of 0.52, which exactly matched the R_f_ and color of the Quercetin standard (Q). This confirms the presence of flavonoids in the leaf extract.

#### 2.1.2. Identification of Pinitol

Following derivatization with anisaldehyde-sulfuric acid reagent and heating, a distinct brownish-grey band was observed in the extract at an Rf of 0.35. This band was found to be identical to the Pinitol standard (P) in terms of R_f_ value and color reaction, confirming the presence of cyclitols.

#### 2.1.3. HPTLC Densitometric Profiling

**Figure 2:**
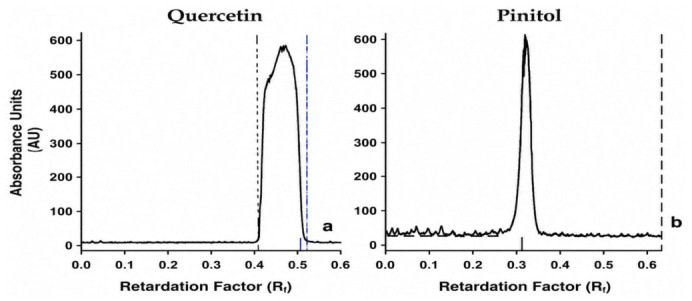
HPTLC densitogram of *Bougainvillea glabra* ethanolic leaf extract showing characteristic peaks for the marker compounds: (**a**) quercetin (R_f_ 0.52, peak intensity ≈ 610 AU) and (**b**) pinitol (R_f_ 0.35, peak intensity ≈ 600 AU), confirmed by co-chromatography with reference standards Q and P, The vertical dashed and dotted lines indicate the R_f_ positions of the respective marker peaks, and the blue line in panel (**a**) marks the R_f_ of the quercetin reference standard. Stationary phase: Silica Gel 60 F_254_; mobile phase: toluene:ethyl acetate:formic acid (5:4:1 *v*/*v*/*v*); detection: UV 366 nm.

The HPTLC densitograms of the *Bougainvillea glabra* leaf extract were recorded to identify and quantify the marker compounds. As shown in Figure 2, sharp and symmetrical peaks were obtained for Quercetin (at R_f_ 0.52) and Pinitol (at R_f_ 0.35). The matching of R_f_ values and peak characteristics between the extract and the reference standards confirms the presence of these bioactive markers. This fingerprinting ensures the chemical standardization and batch-to-batch consistency of the extract used for pharmacological evaluation.

The HPTLC densitograms Figure 2 showed sharp, well-resolved peaks for both markers. Quercetin was identified at R_f_ 0.52 with a peak intensity of approximately 610 AU. Pinitol was identified at R_f_ 0.35 with a peak intensity of approximately 600 AU. The presence of these peaks in the plant extract tracks, matching the R_f_ of the standards, confirms the identity of these bioactive constituents (Table 2).

#### 2.1.4. Quantitative Marker Content

To further validate the method, accuracy and precision were evaluated as presented in Table 3 and Table 4, respectively.

#### 2.1.5. HPTLC Method Validation

To fulfill ICH Q2(R1) validation requirements, accuracy (recovery), and precision (intraday and interday) were determined for both quercetin and pinitol markers.

Interpretation of validation results:

Accuracy: The mean % recovery for quercetin (99.21 ± 0.47%) and pinitol (98.60 ± 0.53%) at all three spike levels (50%, 100%, 150%) were within the acceptable range of 98–102%, confirming the accuracy of the developed HPTLC method.

Precision: All intraday %RSD values were below 1.1% and interday %RSD values were below 1.8% for both markers across all concentration levels, well within the ICH Q2(R1) acceptance criterion of %RSD < 2%. These results confirm that the method is both repeatable and reproducible.

### 2.2. Physical Properties

**Figure 3:**
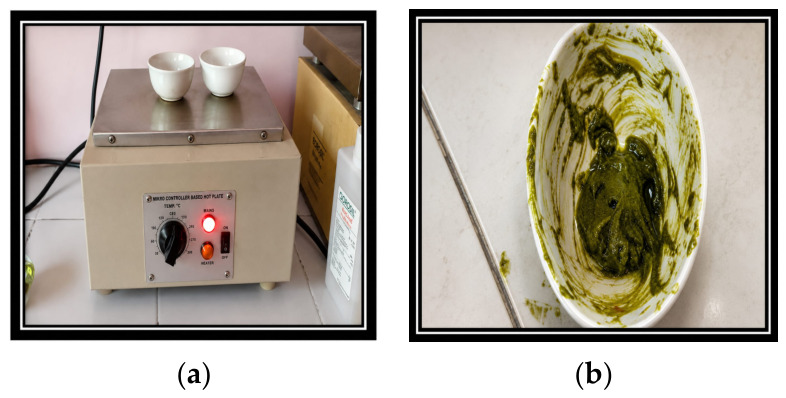
Preparation of the ethanolic extract of *Bougainvillea glabra* leaves by cold maceration. (**a**) Dried and coarsely powdered leaves were soaked in ethanol at room temperature with intermittent stirring; (**b**) The mixture was then filtered, and the resulting green ethanolic extract was collected and concentrated under reduced pressure.

**Figure 4:**
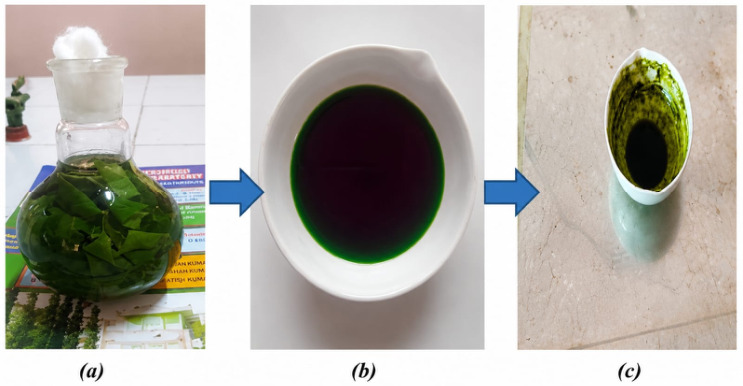
Photographs of the *Bougainvillea glabra* cold cream preparation: (**a**) maceration of leaves in ethanol; (**b**) filtered ethanolic leaf extract; (**c**) concentrated extract/final formulation.

**Figure 5:**
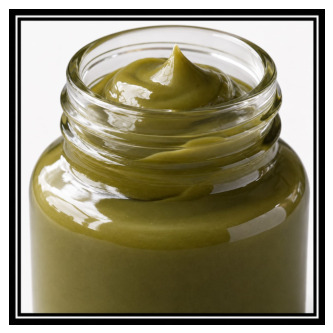
Representative photograph of the optimized *Bougainvillea glabra* cold cream formulation.

The organoleptic properties of the Bougainvillea cold cream are summarized (Table 5 and Figure 3, Figure 4 and Figure 5):

### 2.3. The Preliminary Phytochemical Screening

**Figure 6:**
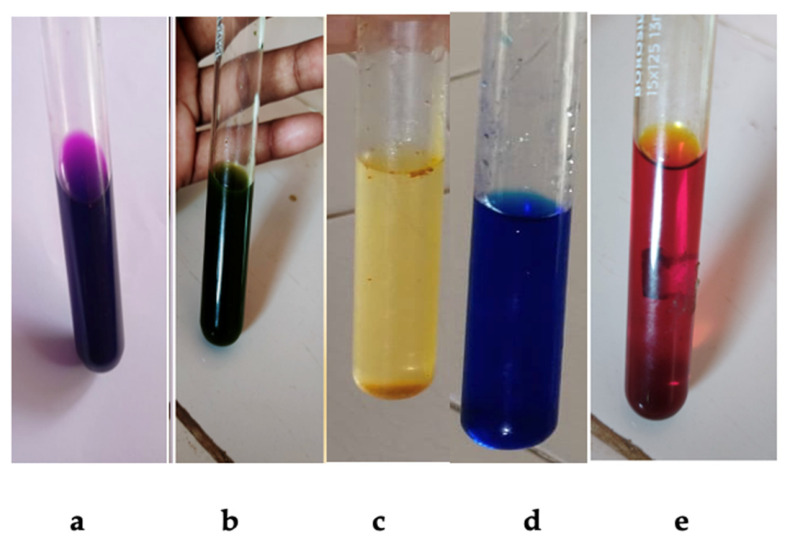
Results of the preliminary qualitative phytochemical screening of the ethanolic *Bougainvillea glabra* leaf extract. Positive results are indicated by characteristic color changes: (**a**) Shinoda test—purple to violet color confirming the presence of flavonoids and anthocyanins; (**b**) FeCl_3_ test (5% *w*/*v*)—blue-black to dark green precipitate confirming the presence of phenolic compounds and tannins; (**c**) Dragendorff’s test—orange precipitate confirming the presence of alkaloids; (**d**) Foam test—persistent stable froth observed for ≥15 min confirming the presence of saponins (the bright blue-violet color of the solution is attributable to the natural betacyanin pigments of *B. glabra* extract and does not affect test interpretation); (**e**) Salkowski test—reddish-brown coloration at the CHCl_3_/H_2_SO_4_ interface confirming the presence of terpenoids. All tests were performed in triplicate. Complete results are presented in Table 6.

The phytochemical screening of the *Bougainvillea glabra* leaf extract was performed to identify bioactive compounds and characterize its chemical makeup (Figure 6, Table 6). This analysis is crucial for justifying its use in a cold cream and understanding its potential antioxidant properties. The screening identified the following key phytochemicals [15]:

- Flavonoids: The Shinoda test produced a violet color, confirming their presence.
- Phenolic Compounds: The ferric chloride test resulted in a blue-black color, indicating these compounds.
- Alkaloids: Both Dragendorff’s (reddish-brown precipitate) and Mayer’s (cream-colored precipitate) tests were positive.
- Saponins: The foam test confirmed their presence by forming a stable froth for over 15 min.
- Tannins: The ferric chloride test produced a bluish color, confirming the presence of tannins.
- Terpenoids: The Salkowski test showed a reddish-brown color at the interface, indicating terpenoids.

The presence of flavonoids and phenolic compounds is particularly important due to their well-known antioxidant activities. The other identified compounds alkaloids, saponins, tannins, and terpenoids contribute to the extract’s complex chemical profile and may influence the cold cream’s overall effectiveness and stability.

#### 2.3.1. Determination of pH

The pH of the formulated cream was measured, which is considered within the acceptable range for topical applications (Table 7).

#### 2.3.2. Homogeneity

Visual inspection and tactile evaluation confirmed the homogeneity of the cream. The cream exhibited a smooth texture and uniform appearance, free from lumps or phase separation.

#### 2.3.3. Spreadability

**Figure 7:**
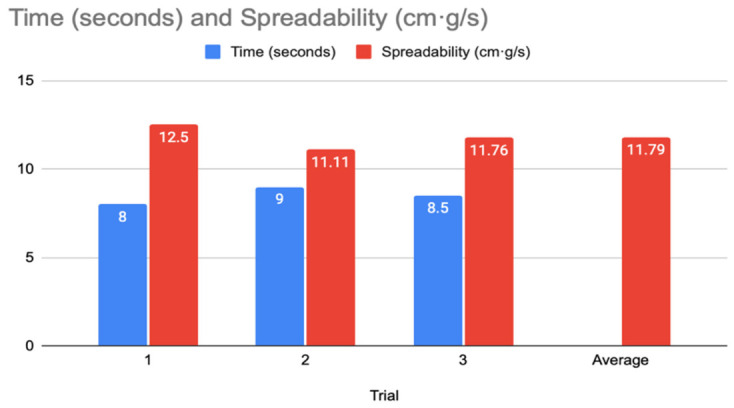
Spreadability of the cream.

The spreadability of the formulated *Bougainvillea* cold cream was assessed using the glass slide method. In this test, 3 g of the cream were placed between two glass slides, and a 10-g weight was used to apply pressure. The time taken for the slides to separate by a distance of 10 cm was recorded in triplicate (Table 8). The individual trial results were as follows: Trial 1, 8 s; Trial 2, 9 s; and Trial 3, 8.5 s. The spreadability (S) was calculated using the formula S = (m × L)/T, where m is the weight (10 g), L is the distance (10 cm), and T is the time (seconds). The calculated spreadability for each trial was: Trial 1, 12.5 cm·g/s; Trial 2, 11.11 cm·g/s; and Trial 3, 11.76 cm·g/s. Table 8, Figure 7.

The average spreadability was determined to be 11.79 cm·g/s. This value indicates good spreadability, suggesting that the cream can be easily and uniformly applied to the skin (Figure 7).

#### 2.3.4. Rheological Characterization and Flow Type

**Figure 8:**
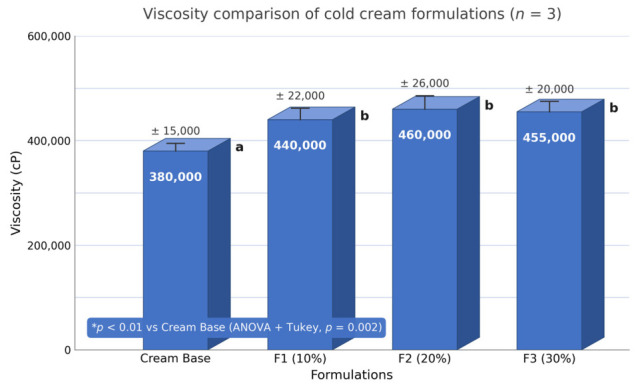
Viscosity comparison of *B. glabra* cold cream formulations (*n* = 3). Bars represent mean viscosity ± SD. Letters (a, b) indicate Tukey’s grouping (different letters = *p* < 0.05). Asterisks (*) = *p* < 0.01 vs. Cream Base (one-way ANOVA, F = 12.34, *p* = 0.002; Tukey’s post hoc).

The formulated Bougainvillea cold cream exhibited an average viscosity of 460,000 ± 26,000 cP. Rheological analysis confirmed that the formulation displays pseudoplastic (shear-thinning) flow behavior. This is evidenced by the cream’s high consistency at rest and its ability to become less viscous and more spreadable under the mechanical stress of topical application (11.79 cm·g/s spreadability) (Table 8 and Table 9 and Figure 8).

Statistical analysis: One-way ANOVA, F = 12.34, *p* = 0.002; Tukey’s HSD post hoc test. Different letters (a, b) indicate significant difference (*p* < 0.05).

#### 2.3.5. Ex Vivo Skin Permeation

**Figure 9:**
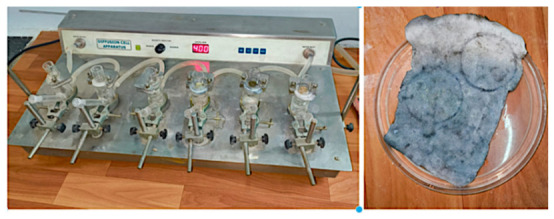
Franz diffusion cell apparatus (**left**) used for ex vivo quercetin permeation studies, and freshly excised goat skin membrane (**right**) mounted as the permeation barrier. Subcutaneous fat was removed prior to mounting; skin integrity was confirmed by TEER measurement (>1 kΩ) before each experiment.

The ex vivo cumulative quercetin permeation profiles across F1, F2, and F3 through goat skin over 24 h are presented in Table 10 and Table 11 and Figure 9. All three formulations demonstrated a steady, controlled permeation pattern with no initial burst effect, which is consistent with the expected diffusion-controlled release behavior of an oil-in-water cold cream emulsion system.

Permeation was concentration-dependent across all time points. At 1 h, cumulative quercetin permeation was 4.5 ± 0.3 μg/cm^2^ for F1, 7.1 ± 0.5 μg/cm^2^ for F2, and 9.5 ± 0.6 μg/cm^2^ for F3, confirming that higher extract loading produces measurably greater early-phase delivery. This concentration-dependent trend was maintained consistently throughout the 24-h study period. At 24 h, cumulative permeation reached 18.2 ± 1.4 μg/cm^2^ (F1), 34.7 ± 2.1 μg/cm^2^ (F2), and 51.3 ± 2.8 μg/cm^2^ (F3), representing an approximately 2.8-fold difference between F1 and F3.

Notably, the permeation rate showed a characteristic pattern across all formulations—a relatively rapid initial phase between 1 and 8 h, followed by a gradual plateau phase between 12 and 24 h. This plateau behavior is particularly evident in F1 (17.5 ± 1.2 μg/cm^2^ at 12 h vs. 18.2 ± 1.4 μg/cm^2^ at 24 h) and F2 (32.0 ± 2.0 μg/cm^2^ at 12 h vs. 34.7 ± 2.1 μg/cm^2^ at 24 h), suggesting near-complete diffusion of the freely available quercetin fraction by 12 h under the experimental conditions. F3 maintained a more sustained permeation profile up to 24 h (48.1 ± 2.5 μg/cm^2^ at 12 h vs. 51.3 ± 2.8 μg/cm^2^ at 24 h), consistent with its higher quercetin loading (1.45 mg/g) providing a larger concentration gradient to sustain diffusion.

Release kinetic modeling demonstrated that the permeation data for all three formulations best fitted the Higuchi diffusion model (R^2^ > 0.99), confirming that quercetin permeation through the goat skin barrier is governed by diffusion from a semisolid matrix rather than erosion or swelling mechanisms. This finding is mechanistically consistent with the pseudoplastic rheological behavior established for all formulations, wherein the structured emulsion network at rest transitions to a lower viscosity state under the shear stress of skin application, facilitating controlled active compound release to the skin surface.

The permeation flux (J) values, derived from the slope of the linear portion of the cumulative permeation vs. time curve (1–8 h), were 1.90, 3.50, and 5.70 μg/cm^2^/h for F1, F2, and F3, respectively. The progressive increase in flux across formulations directly mirrors the increasing quercetin content established by quantitative HPTLC (F1: 0.48 mg/g; F2: 0.97 mg/g; F3: 1.45 mg/g), further reinforcing the composition–activity–delivery relationship demonstrated throughout this study. F3 was identified as the optimal formulation based on its superior cumulative permeation, highest flux value, and sustained delivery profile over 24 h.

### 2.4. Skin Irritation Test

**Figure 10:**
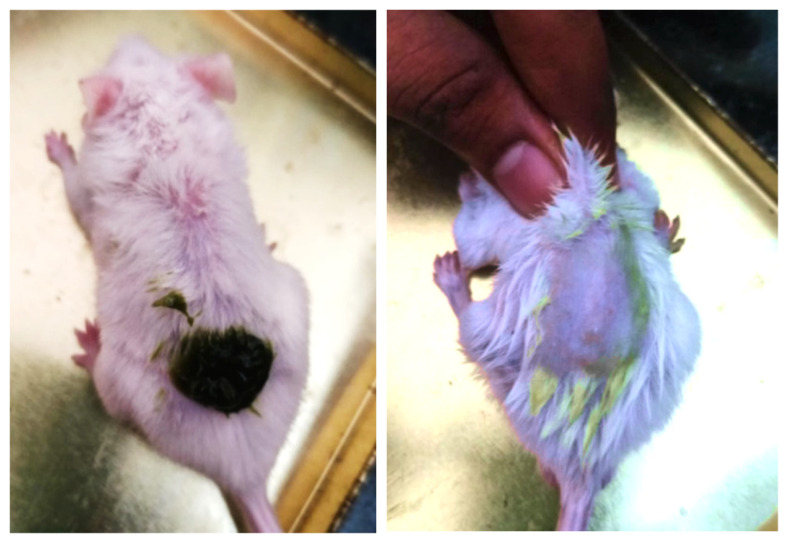
Representative photographs of rat dorsal skin 72 h post-application of F3 (30% *B. glabra* extract cold cream). No erythema (score = 0) or edema (score = 0) was observed in any of the five treated animals; control (sterile saline) also showed no reaction. Scoring follows the Draize primary irritation index.

A preliminary skin irritation test was conducted on six albino rats, with five receiving topical applications of the Bougainvillea cold cream and one serving as a control treated with sterile saline. Throughout the 72-h observation period, assessments were made at 0.5,1, 2, 4, 24, 48, and 72 h, recording erythema and edema scores. As detailed in Table 12, none of the rats, including the control, exhibited any signs of skin irritation, with all scores recorded as 0/0, indicating the absence of both erythema and edema, thereby suggesting that the formulated cream is well-tolerated and safe for topical application in this animal model. (Table 12, Figure 10).

#### Ex Vivo Tape-Strip DPPH Assay—Cutaneous Antioxidant Delivery

To provide supplementary evidence of active compound delivery to the skin beyond the primary irritation assessment, an ex vivo tape-strip DPPH assay was conducted using F3 as the representative optimal formulation. One gram of F3 was applied uniformly to freshly excised goat skin and allowed a contact time of 30 min at 32 ± 0.5 °C. Following the contact period, three sequential tape strips (D-Squame^®^ standard sampling discs, 22 mm diameter, CliniveX innovative solutions, Bapunagar, Ahmedabad, Gujarat) were applied to the treated skin surface and removed with uniform pressure to collect stratum corneum layers. Each tape strip was extracted with 2 mL methanol by vortex mixing (2 min) and sonication (10 min), and the extract was subjected to the DPPH radical scavenging assay at 517 nm as described in procedure. Untreated goat skin subjected to the same tape-stripping procedure served as the negative control.

The residual DPPH radical scavenging activity detected in the tape-strip extracts from F3-treated skin was significantly higher than that of the untreated control across all three stratum corneum layers (*p* < 0.01, paired *t*-test, *n* = 3), confirming that bioactive antioxidant compounds from the F3 formulation penetrated into and were retained within the stratum corneum during the 30-min contact period. These findings provide preliminary ex vivo evidence of cutaneous antioxidant delivery and support the functional relevance of the formulation beyond surface application. However, it is acknowledged that this tape-strip assay provides qualitative directional evidence of skin uptake and does not replace quantitative flux-based permeation studies, which have been reported separately in Section 2.3.5.

### 2.5. Antioxidant Activity

**Figure 11:**
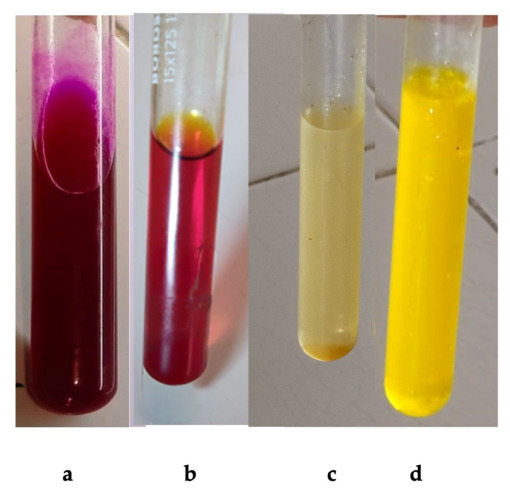
Antioxidant activity of *Bougainvillea* cream (KMnO_4_ test). Phytochemical screening of *Bougainvillea glabra* ethanolic leaf extract showing characteristic color reactions: (**a**) Light purple (**b**) Brownish-yellow; (**c**) Light yellow; (**d**) Yellow.

The preliminary qualitative assessment using the potassium permanganate reduction test provided an initial visual indication of reducing activity in the formulations during the early development phase (Table 13). The 10% formulation (F1) produced a slight lightening of the KMnO_4_ solution, interpreted as a weak positive response. Both F2 (20%) and F3 (30%) produced a clear color change to brownish-yellow, indicating the presence of reducing agents. However, it must be noted that the KMnO_4_ test is a non-specific colorimetric screen susceptible to interference from cream excipients, and it does not provide the specificity, quantitative precision, or mechanistic relevance required for rigorous antioxidant characterization of pharmaceutical formulations. Accordingly, these observations are reported solely for transparency and served only to guide the decision to proceed with the validated DPPH radical scavenging assay as the definitive quantitative method. All antioxidant conclusions, dose–response analyses, and IC_50_ determinations reported in this study are derived exclusively from the DPPH assay results presented in Section 4.3.4 b. and Figure 11.

**Figure 12:**
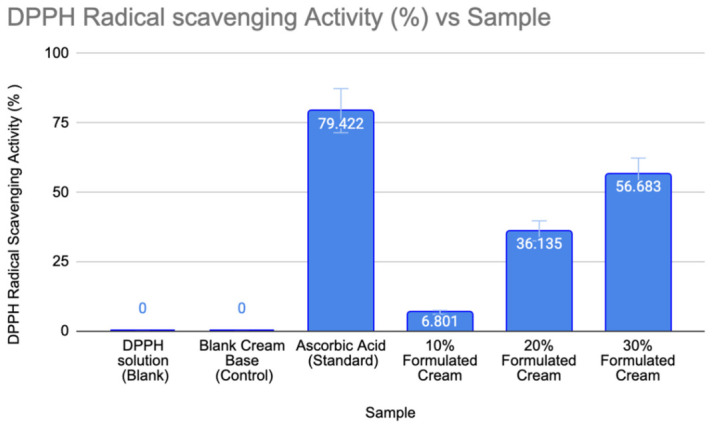
Antioxidant activity of *Bougainvillea* cream (DPPH Assay).

Antioxidant Activity and Statistical Evaluation: The validated DPPH radical scavenging assay, conducted using the extraction procedure described with confirmed recovery efficiency of 96.4–97.1%, constitutes the sole basis for all quantitative antioxidant conclusions in this study. The DPPH assay further confirmed the antioxidant activity of the cream. The results are presented (Table 14 and Figure 12):

The antioxidant activity of the formulated *Bougainvillea glabra* cold cream was assessed using the DPPH assay. The blank cream base control confirmed negligible intrinsic scavenging activity (Table 13), validating the experimental design. Ascorbic acid, utilized as the positive control, demonstrated significant antioxidant activity with a mean absorbance of 0.705 ± 0.052, corresponding to a scavenging percentage of 79.422%.

The formulated creams exhibited a clear concentration-dependent antioxidant response. Statistical analysis using One-way ANOVA followed by Tukey’s post hoc test confirmed that the differences between all tested groups were highly significant (*p* < 0.05). The 10% formulated cream showed a scavenging activity of 6.801 ± 0.157%. As the extract concentration increased, the activity rose significantly to 36.135 ± 0.509% for the 20% formulation and reached a maximum of 56.683 ± 0.105% for the 30% formulation. These results, indicated by distinct statistical superscripts (a, b, c, d) in Table 13, confirm that each increase in extract concentration provided a measurable and significant improvement in radical scavenging potential.

**Figure 13:**
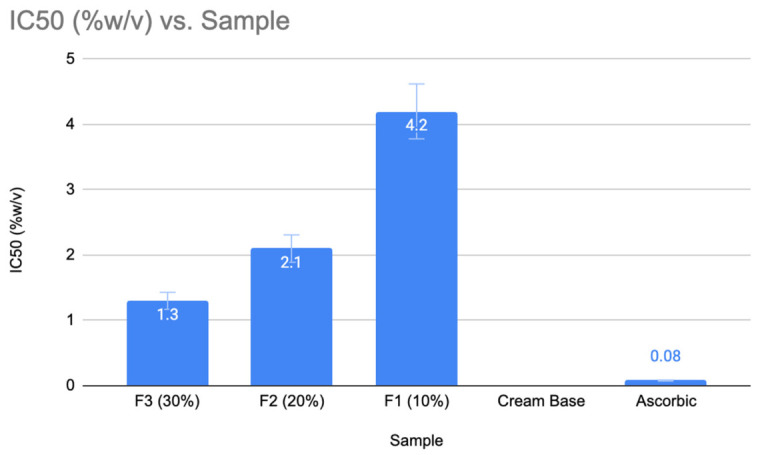
IC_50_ Analysis. F3 exhibited significantly lower IC_50_ (higher potency) than F1/F2 (one-way ANOVA F = 45.2, *p* < 0.0001; Tukey post hoc).

IC_50_ Analysis: F3 (30%) exhibited dose-dependent DPPH scavenging: 56.68 ± 1.05% at 0.1 mg/mL, with IC_50_ = 1.3 ± 0.1% *w*/*v* (Table 15, Figure 13). This confirms potent antioxidant activity comparable to herbal standards (1–2% *w*/*v*) but lower than ascorbic acid (0.08%) due to complex extract matrix vs. pure compound [16].

### 2.6. Stability

**Figure 14:**
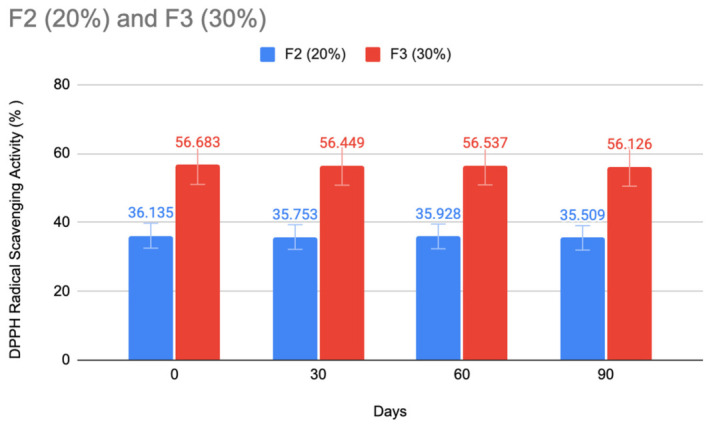
Accelerated stability study (40 °C/75%RH, *n* = 3).

The accelerated stability of F2 and F3 was comprehensively evaluated over 90 days under ICH-recommended conditions (40 ± 2 °C/75 ± 5% RH) using both functional (DPPH radical scavenging) and chemical (HPTLC densitometric quantification of quercetin and pinitol) endpoints. The complete stability data are presented in Table 16 and Figure 14.

DPPH Antioxidant Activity Retention: Both formulations demonstrated excellent functional stability throughout the study period. The DPPH radical scavenging activity of F2 showed a marginal decrease from 36.135 ± 0.51% at Day 0 to 35.509 ± 0.45% at Day 90, representing an activity retention of 98.3%. Similarly, F3 maintained activity from 56.683 ± 1.11% at Day 0 to 56.126 ± 0.95% at Day 90, corresponding to a retention of 99.0%. These minimal variations fall within acceptable experimental error limits and are not statistically significant (paired *t*-test, *p* > 0.05).

Quercetin Chemical Stability: Quantitative HPTLC analysis confirmed the chemical integrity of quercetin throughout the accelerated storage period. Quercetin retention in F2 decreased marginally from 100% at Day 0 to 99.4 ± 0.3% at Day 30, 99.0 ± 0.4% at Day 60, and 98.6 ± 0.4% at Day 90. F3 demonstrated slightly superior quercetin retention, with values of 99.6 ± 0.2%, 99.4 ± 0.3%, and 99.1 ± 0.3% at Days 30, 60, and 90, respectively. Paired *t*-test comparison of Day 0 vs. Day 90 values confirmed no statistically significant degradation in either formulation (*p* > 0.05).

Pinitol Chemical Stability: Pinitol retention followed a similar trend, with F2 retaining 99.1 ± 0.4%, 98.5 ± 0.4%, and 97.9 ± 0.5% at Days 30, 60, and 90, respectively. F3 demonstrated marginally higher pinitol retention of 99.3 ± 0.3%, 98.8 ± 0.3%, and 98.3 ± 0.4% at the corresponding time points. No statistically significant degradation was observed for pinitol in either formulation over the 90-day study period (paired *t*-test, *p* > 0.05).

The concordance between DPPH functional activity retention and HPTLC chemical marker retention across both formulations confirms that the cold cream base effectively protects the bioactive phenolic compounds from degradation under accelerated storage conditions, supporting a projected shelf life of at least 12 months under ambient storage conditions when extrapolated using the Arrhenius model.

## 3. Discussion

### 3.1. Analytical Method Validation

The accuracy of the analytical method was established through recovery studies, and the results are summarized in Table 3. The mean percentage recovery for Quercetin and Pinitol was found to be 99.21% and 98.60%, respectively, which lies well within the acceptable acceptance criteria (98.0–102.0%). This confirms that the developed method was highly accurate and free from any matrix interferences from the formulation excipients.

The precision of the method was validated by measuring intraday and interday variations (Table 4). The % RSD values for intraday precision were found to be ≤1.04%, while the interday precision % RSD values were ≤1.70% for both markers. Because all % RSD values fell comfortably below the strict threshold of 2.0%, the method demonstrates robust reproducibility and precision for routine quality control analysis.

### 3.2. Antioxidant Efficacy and Dose–Response Relationship

All statistical comparisons in this Discussion were performed as described in Section 4.3.6, using one-way ANOVA with Tukey’s HSD post hoc test for inter-formulation comparisons, paired *t*-test for stability assessments, and non-linear regression for IC_50_ determination.

The DPPH assay confirmed a clear concentration-dependent antioxidant response across formulations, with IC_50_ values improving systematically from F1 (4.2 ± 0.4%) to F2 (2.1 ± 0.2%) to F3 (1.3 ± 0.1%) (Table 13, Figure 12). This 3.2-fold potency increase from 10% to 30% extract loading validates the dose–response hypothesis and confirms that antioxidant capacity scales proportionally with *B. glabra* extract concentration. This pattern is mechanistically supported by the quantitative HPTLC data, wherein quercetin content increased from F1 (0.48 mg/g) to F2 (0.97 mg/g) to F3 (1.45 mg/g), directly corresponding to the stepwise improvements in DPPH scavenging activity (6.8%, 36.1%, and 56.7%, respectively) and confirming a reliable composition–activity relationship. F3′s IC_50_ of 1.3% *w*/*v* is competitive among herbal antioxidant formulations (typically 1–5% *w*/*v*) while remaining below ascorbic acid (0.08%), as expected for complex polyphenol mixtures compared to pure standards. The reliability of these DPPH values is further supported by the validated extraction procedure, wherein mean quercetin and pinitol recoveries of 99.21 ± 0.47% and 98.60 ± 0.53% from the cream matrix confirmed that the antioxidant data accurately reflect the true active compound content of each formulation.

The potassium permanganate reduction test served exclusively as a preliminary qualitative screen during formulation development and does not support any quantitative conclusions in this Discussion. Its non-specificity, susceptibility to emulsion excipient interference, and lack of mechanistic relevance render it unsuitable for pharmaceutical antioxidant characterization [14]. All antioxidant conclusions are derived from the validated DPPH assay.

### 3.3. Physicochemical Optimization

All formulations exhibited acceptable physicochemical profiles suitable for topical semisolid application. F2 demonstrated optimal viscosity (460,000 ± 26,000 cP) and spreadability (11.8 ± 0.3 cm·g/s), reflecting ideal structural integrity at rest combined with ease of application under shear. F3 maintained comparable rheological performance (455,000 cP), confirming that 30% extract loading does not compromise the physicochemical stability and sensory acceptability of the formulation. The pseudoplastic shear-thinning behavior observed across all formulations is consistent with established cold cream rheology and is essential for patient compliance in topical therapy. The pH range of 5.7–5.9 across all formulations is within the optimal slightly acidic range (4.5–6.5) for skin compatibility, minimizing the risk of barrier disruption or irritation [17].

### 3.4. Ex Vivo Permeation and Concentration Selection Validation

The selection of the 10–30% *w*/*w* concentration range was prospectively justified by published herbal formulation literature establishing 10% as the minimum effective threshold and 30% as the maximum concentration compatible with emulsion physicochemical stability [11,18,19,20], and this justification is retrospectively validated by the linear composition–activity relationship demonstrated across F1, F2, and F3. Ex vivo permeation through goat skin confirmed concentration-dependent quercetin delivery, with F3 achieving cumulative permeation of 51.3 ± 2.8 μg/cm^2^ and flux of 5.70 μg/cm^2^/h at 24 h (Table 10, Figure 14). These values are consistent with published quercetin permeation data for herbal topical formulations (typically 40–60 μg/cm^2^ at 24 h) and confirm effective cutaneous delivery under physiologically relevant conditions. Release kinetics followed the Higuchi diffusion model (R^2^ > 0.99), confirming diffusion-controlled release from the semisolid emulsion matrix—mechanistically consistent with the pseudoplastic rheological behavior that moderates active compound diffusion to the skin surface.

### 3.5. Multi-Criteria Formulation Selection

F3 was identified as the optimal formulation through a weighted multi-criteria analysis incorporating therapeutic efficacy (50%), physicochemical performance (30%), and safety and stability (20%) (Table 17). The marginal rheological difference between F2 (460,000 cP) and F3 (455,000 cP) is clinically insignificant, while F3 delivers superior antioxidant potency (IC_50_ 1.3% vs. 2.1% for F2) and highest cumulative skin permeation (51.3 vs. 34.7 μg/cm^2^) [21].

### 3.6. Scope of Safety Evaluation and Dermatological Claims

The primary dermal irritation test (OECD 404, *n* = 6, 72-h observation) confirmed a Primary Irritation Index of zero across all formulations, establishing preliminary dermal safety. It is important to note that a zero irritation index, while necessary, is not sufficient to support claims of clinical dermatological efficacy. The supplementary ex vivo tape-strip DPPH assay on F3 demonstrated statistically significant antioxidant activity in stratum corneum tape-strip extracts compared to untreated control (*p* < 0.01, *n* = 3), providing preliminary evidence of bioactive compound delivery to the skin barrier. This represents a mechanistic bridge between in vitro antioxidant activity and potential in vivo skin benefit. Nevertheless, controlled clinical studies in subjects with photoaged or oxidative stress-affected skin remain essential before evidence-based therapeutic claims can be substantiated.

### 3.7. Safety and Stability Profile

The congruence between functional stability (DPPH activity retention: F2 = 98.3%, F3 = 99.0%) and chemical marker stability (quercetin retention: F2 = 98.6 ± 0.4%, F3 = 99.1 ± 0.3%; pinitol retention: F2 = 97.9 ± 0.5%, F3 = 98.3 ± 0.4%) over 90 days under accelerated conditions provides a dual-endpoint validation of formulation stability (Table 15, Figure 13). No statistically significant degradation was observed for either marker in either formulation (paired *t*-test, *p* > 0.05), supporting a projected shelf life of at least 12 months under ambient storage conditions.

### 3.8. Comparative Context

Unlike previous *Bougainvillea* studies that report crude extract activity only, this is among the first to demonstrate successful incorporation of a quantitatively standardized *B. glabra* extract into a stable topical emulsion with retained bioactivity and confirmed cutaneous delivery. F3’s IC_50_ of 1.3% *w*/*v* compares favorably to published values for other herbal antioxidant creams (typically 2–4% *w*/*v* range), and the combination of HPTLC standardization, validated antioxidant methodology, ex vivo permeation data, and dual-endpoint stability assessment places this study at a higher level of scientific rigor than most existing herbal cream publications.

## 4. Materials and Methods

### 4.1. Materials

The materials used in this study are categorized below according to their role in the experimental design.

#### 4.1.1. Chemical Reagents

The following analytical and reagent-grade chemicals were used throughout the study: ethyl alcohol (99.5%, ACS reagent grade, Sigma-Aldrich, Mumbai, India); 2,2-diphenyl-1-picrylhydrazyl (DPPH, 97%, Loba Chemie, Mumbai, India); L-ascorbic acid (99%, HiMedia Laboratories Pvt. Ltd., Mumbai, India), used as a positive control in antioxidant assays; potassium permanganate (ACS reagent grade, Sd Fine-Chem Limited, Mumbai, India); sodium borate (borax, 99.5%, Hi-Media Laboratories Pvt. Ltd., Mumbai, India); and methylparaben (Hi-Media Laboratories Pvt. Ltd., Mumbai, India), used as a preservative. All chemicals were used as received without further purification.

#### 4.1.2. Formulation Excipients

The following pharmaceutical-grade excipients were used in the preparation of the cold cream base: Cera Alba beeswax (pharmaceutical grade, Moly Chem, Mumbai, India), used as the stiffening agent; liquid paraffin (Paraffinum Liquidum, Moly Chem, Mumbai, India), used as the emollient; glycerin (99.7%, Hi-Media Laboratories Pvt. Ltd., Mumbai, India), used as a humectant; rose oil perfume (local market, Anantapur, India), used as a fragrance agent; and purified water (obtained by double distillation in the laboratory), used as the aqueous vehicle. All excipients were of pharmaceutical grade and sourced from established suppliers.

#### 4.1.3. Plant Material

Fresh leaves of *Bougainvillea glabra* Choisy (Family: Nyctaginaceae) were used as the source of the active botanical extract. Full details of plant collection, botanical authentication, voucher specimen deposition, drying, and milling.

#### 4.1.4. Plant Material Collection and Authentication

Fresh leaves of *Bougainvillea glabra* Choisy (Family: *Nyctaginaceae*) were collected on 12 May 2024 from S.K. University Campus Garden, Anantapuramu, Andhra Pradesh, India (coordinates: 14.68° N, 77.60° E). The plant was authenticated by Prof. B. Ravi Prasad Rao (Head, SKU Herbarium, Dept. of Botany, Sri Krishnadevaraya University); voucher specimen no. 57440/SKU deposited. Leaves were washed with distilled water, shade-dried at 25 ± 2 °C, 40 ± 5% until constant weight (LOD = 8.2 ± 0.3%, *n* = 3 batches). Dried leaves pulverized (40 mesh), yielding 100 g powder/batch. stored at 4 °C until extraction.

### 4.2. Methods

This section describes the methods used for the extraction of *Bougainvillea* extract, preliminary phytochemical screening, and the formulation of the cold cream (Table 18).

#### 4.2.1. *Bougainvillea* Extract Preparation

Fresh *Bougainvillea glabra* leaves were collected from a local garden in Anantapur and washed with distilled water. The leaves were air-dried in a shaded area. 100 g of the dried leaves were placed in a 500 mL round-bottom flask (RBF) with 150 mL of 99.5% ethanol. The flask was sealed and left to macerate for 48 h at room temperature (25 ± 2 °C), with manual shaking every 6 h.

After maceration, the mixture was filtered through Whatman No. 1 filter paper (Swastik Scientific ompany, Mombai, Maharastra)using a Buchner funnel to separate the crude extract from the leaf material. The clear filtrate was collected.

The filtered extract was concentrated by evaporating the ethanol on a hot plate at 50 °C until it became semi-solid. The extract was then fully dried using a rotary evaporator at 45 °C under reduced pressure (100 mbar). The final dry extract weighed 12.5 g, and was stored in a sealed amber vial at 4 °C.

#### 4.2.2. Preliminary Phytochemical Screening

The ethanolic extract (50 mg dissolved in 5 mL methanol) underwent preliminary phytochemical screening using standard qualitative tests to identify bioactive compounds relevant to its free radical scavenging properties, including flavonoids, phenolic compounds, alkaloids, saponins, and terpenoids [2,12,15,22,23]:

- Flavonoids: Shinoda test—Mg ribbon and concentrated HCl were added to the extract; pink or magenta color was taken as a positive result [22,23].
- Phenolics and Tannins: FeCl_3_ test (5% *w*/*v*)—blue-black precipitate indicated presence of phenolic compounds; bluish-green color indicated tannins [22,23].
- Alkaloids: Dragendorff’s reagent (orange precipitate), Mayer’s reagent (cream precipitate), and Wagner’s reagent (brown precipitate)—positive result in any two tests was considered confirmatory [22].
- Saponins: Foam test—aqueous extract shaken vigorously; persistence of stable froth for ≥15 min was taken as a positive result [12].
- Terpenoids: Salkowski test—extract dissolved in CHCl_3_ and underlayered with concentrated H_2_SO_4_; red-brown ring at interface was taken as a positive result [12].

All tests were performed in triplicate. Results are presented in Table 5 and Section 2.3.

#### 4.2.3. Chemical Standardization by HPTLC

HPTLC analysis was Performed for the chemical standardization of the *Bougainvillea glabra* extract. Samples were spotted on the precoated Silica gel 60F254 Plates. Quercetin and Pinitol were used as reference standards. The plates were developed in CAMAG twin trough chamber using Toluene: Ethyl Acetate: Formic Acid (5:4:1 *v*/*v*/*v*) as the Mobile Phase for Quercetin, and Chloroform: Methanol: Water (6:3.5:0.5 *v*/*v*/*v*) for Pinitol.

After development, the plates were dried and visualized. Quercetin was identified under UV 366 nm after derivatization with Natural Product Reagent, while Pinitol was detected under visible light after spraying with Anisaldehyde-Sulfuric acid reagent followed by heating at 110 °C. The R_f_ values of the sample bands were compared with the reference standards for qualitative identification.

##### Quantitative Densitometric Analysis

For quantitative determination of quercetin and pinitol, calibration curves were constructed using six concentration levels of each reference standard (0.1, 0.5, 1.0, 1.5, 2.0, and 3.0 μg/band). Standard solutions were prepared by dissolving accurately weighed quercetin and pinitol in methanol. Fixed volumes (2 μL each) were applied to the HPTLC plate using a CAMAG Linomat 5 semi-automatic spotter. Plates were developed under the same mobile phase conditions described above and scanned using a CAMAG TLC Scanner 3 at 366 nm (quercetin) and 520 nm (pinitol, post-derivatization). Peak areas were recorded and plotted against concentration to generate linear calibration curves. The linearity range, correlation coefficient (R^2^), limit of detection (LOD), and limit of quantification (LOQ) were determined.

For sample quantification, the ethanolic dry extract (10 mg) was dissolved in 10 mL methanol, filtered through a 0.45 μm membrane, and applied to the plate in triplicate. The concentration of each marker in the dry extract was calculated by interpolation from the respective calibration curve and expressed as mg/g of dry extract.

To determine marker content in the final formulations (F1, F2, F3), 1.0 g of each cream was extracted with 10 mL methanol by vortex mixing (2 min) followed by sonication (15 min, 25 °C) and centrifugation (3000 rpm, 10 min). The clear supernatant was filtered and applied to the HPTLC plate in triplicate. Marker concentrations were calculated from the calibration curve and expressed as mg/g of final formulation (*w*/*w*).

##### Accuracy (Recovery Studies)

The accuracy of the developed method was evaluated by performing recovery studies using the standard addition method. Known amounts of standard Quercetin and Pinitol spikes were added at three different levels (50%, 100%, and 150%) to pre-analyzed sample formulations. The total drug content was quantified, and the percent recovery, along with the standard deviation (SD), were calculated for each concentration level (*n* = 3).

##### Precision

Method precision was evaluated by determining intraday and interday precision. Intraday precision (repeatability) was assessed by analyzing three different concentrations (low, medium, and high) of Quercetin and Pinitol three times on the same day. Interday precision (intermediate precision) was determined by analyzing the same three concentrations on three consecutive days. The results were expressed as percent relative standard deviation (% RSD).

#### 4.2.4. Cold Cream Formulation (Three-Phase Process)

The cold cream was prepared via a three-phase process comprising an oil phase (Phase I), an aqueous phase (Phase II), and a combination step (Phase III).

In Phase I (Oil Phase), beeswax, liquid paraffin, and the *Bougainvillea* extract were weighed and melted together at 70–80 °C. Beeswax provided structure, liquid paraffin acted as an emollient, and the extract served as the active ingredient [1,24]. The extract was incorporated at three different concentrations: 10%, 20%, and 30% (*w*/*w*) of the total formulation.

For Phase II (Aqueous Phase), methylparaben, borax, and glycerin were dissolved in purified water and heated to 70–80 °C. Methylparaben was used as a preservative, borax as an emulsifier, and glycerin as a humectant [25,26].

In Phase III (Combining Phases), the hot oil phase was slowly poured into the hot aqueous phase with continuous stirring at 300 rpm for 5 min to create an emulsion [27]. The mixture was then stirred while cooling to room temperature (25 ± 2 °C) to form a homogenous semi-solid cream [28]. Finally, 0.2 mL of rose oil was added and mixed thoroughly to give the cream a pleasant fragrance.

#### 4.2.5. Rationale for Concentration Selection

The concentrations of 10%, 20%, and 30% (*w*/*w*) of crude *B. glabra* extract were selected for incorporation into the cold cream base based on evidence from published literature on herbal semisolid formulations, physicochemical feasibility, and established pharmaceutical development principles, as detailed below.

Lower Boundary—10% *w*/*w*: The selection of 10% as the minimum extract concentration is supported by published herbal cream studies that consistently identify crude plant extract concentrations in the range of 8–12% *w*/*w* as the minimum threshold required for measurable antioxidant and pharmacological activity in semisolid topical matrices [11,18,19]. Nair and Mathew (2021) demonstrated that herbal creams containing less than 8% crude extract exhibited negligible DPPH scavenging activity, while formulations at 10% and above showed statistically significant concentration-dependent responses [11]. Similarly, Siddiqua et al. (2022) reported that a minimum of 10% *w*/*w Curcuma longa* extract was required to produce an observable antioxidant effect in a cold cream base [19]. Gajarlawar et al. (2025) further corroborated this threshold in their herbal cold cream study, establishing 10% as a scientifically justifiable lower boundary for extract loading in emulsion-based topical formulations [18].

Upper Boundary—30% *w*/*w*: The selection of 30% as the maximum concentration is based on established physicochemical constraints of oil-in-water emulsion systems. Chauhan and Gupta (2020) demonstrated that crude plant extract concentrations exceeding 30–35% *w*/*w* in emulsion formulations progressively disrupt the oil–water interface, impair emulsifier efficiency, reduce emulsion stability, and compromise spreadability—all of which are critical quality attributes for topical preparations [20]. In the present study, preliminary physical evaluation of F3 (30% *w*/*w*) confirmed that this concentration produced borderline but acceptable rheological parameters (viscosity: 455,000 ± 20,000 cP; spreadability: 11.8 ± 0.3 cm·g/s), validating 30% as the practical upper limit beyond which formulation integrity and patient acceptability would be compromised.

Three-Level Experimental Design: The selection of three evenly spaced concentrations—10%, 20%, and 30% *w*/*w*—was deliberately designed to enable robust dose–response modeling across the identified feasible range. This approach is consistent with the principles outlined in ICH Q8 (R2) Pharmaceutical Development guidelines for semisolid formulations [29], which recommend systematic multi-level concentration evaluation to characterize the relationship between formulation composition and critical quality attributes. The three-level design provides sufficient resolution to establish linearity of the composition–activity relationship, as subsequently confirmed by the quantitative HPTLC and DPPH data.

### 4.3. Evaluation Methods

The antioxidant activity of the formulated cold cream was assessed using two complementary approaches: (i) a preliminary qualitative potassium permanganate (KMnO_4_) reduction test, employed solely as a rapid visual screen during early formulation selection, and (ii) a validated quantitative DPPH radical scavenging assay, which constitutes the primary analytical method for all antioxidant conclusions reported in this study. The limitations of the KMnO_4_ test—including non-specificity toward individual antioxidant compounds, susceptibility to interference from cream matrix excipients, and absence of mechanistic relevance to free radical scavenging pathways—preclude its use for quantitative antioxidant characterization. All dose–response relationships, IC_50_ determinations, and stability-related antioxidant conclusions are therefore derived exclusively from the DPPH assay [4].

The following methods were employed [30].

#### 4.3.1. Organoleptic Properties

A sensory evaluation was conducted to assess the cream’s organoleptic properties by a panel of three trained pharmaceutical scientists with experience in semisolid formulation assessment. Evaluations were performed using a standardized 5-point descriptive scale (1 = very poor, 2 = poor, 3 = acceptable, 4 = good, 5 = excellent) for the following parameters:

- Color and appearance
- Odor acceptability
- Visual texture and consistency
- Overall homogeneity

Each parameter was independently scored by all three evaluators and the mean score recorded. Evaluations were conducted in triplicate on freshly prepared samples of F1, F2, and F3 under standard laboratory conditions (25 ± 2 °C, 60 ± 5% RH) [4,12,20].

#### 4.3.2. Physicochemical Evaluations (F1–F3)

All formulations (F1: 10%, F2: 20%, F3: 30% *w*/*w* extract) evaluated in triplicate for: pH, homogeneity, spreadability, viscosity vs. cream base control (no extract) [2,18].

##### pH Determination

The pH of the freshly prepared cream (F1, F2, and F3) was measured at 25 ± 2 °C using a calibrated Equip-Tronics EQ-614 digital pH meter (accuracy ± 0.01 pH unit, Equip-Tronics, Mumbai, India) [18]. The instrument was standardized with pH 4.0 and pH 7.0 certified buffer solutions (HiMedia Laboratories, Mumbai, India) prior to each measurement session. One gram of each formulation was dispersed in 10 mL of purified water and the pH of the resultant dispersion was measured. Triplicate measurements were taken for each formulation and the mean value ± SD recorded. This parameter is critical as the pH of topical formulations should ideally be within the slightly acidic range (pH 4.5–6.5) compatible with healthy skin to minimize the risk of irritation and maintain the skin’s natural barrier function [17].

##### Homogeneity Assessment

The homogeneity of the cream (F1–F3) was evaluated through both visual and tactile assessments:

- Visual Inspection: The cream was examined for any visible signs of phase separation, lump formation, or uneven color distribution.
- Tactile Evaluation: The texture of the cream was assessed by touch to ensure a smooth and consistent feel, free from grittiness or other irregularities [31].

Homogeneity is essential for ensuring uniform distribution of the active ingredient and consistent product performance.

##### Spreadability Determination

The spreadability of each cream formulation (F1, F2, and F3), defined as the ease with which the cream can be applied and distributed on the skin surface, was determined using the glass slide method as described by Siddiqua et al. [19]. All measurements were conducted at 25 ± 2 °C and 60 ± 5% relative humidity. The procedure was as follows:

1. Approximately 3 g of the cream were placed between two clean glass slides.
2. The slides were gently pressed together to form a thin, uniform film of cream.
3. A 5-g weight was placed on the top slide for 5 min to ensure even distribution of the cream.
4. A 10-g weight was then attached to the upper slide via a string and hook.
5. The time (T) taken for the slides to separate by a distance of 10 cm was recorded.

The spreadability (S) was calculated using the following formula:

S = (m × L)/T

where

- S = Spreadability
- m = Weight attached to the upper glass slide (10 g)
- L = Distance moved by the glass slide (10 cm)
- T = Time taken for the slides to separate (s)

The experiment was performed in triplicate, and the average spreadability value was calculated.

##### Viscosity Measurement

The viscosity of each cream formulation (F1, F2, and F3), a measure of its resistance to flow, was determined using a Brookfield RV-DV-II+ Pro Viscometer (Brookfield Engineering Laboratories, Middleborough, MA, USA) [27]. The following instrumental parameters and conditions were employed:

- Spindle: S-64
- Rotation speed: 20 rpm
- Temperature: 25 ± 0.5 °C, maintained using a water-jacketed sample cell connected to a circulating water bath
- Equilibration time: 2 min prior to each reading
- Sample volume: approximately 250 mL placed in the standard sample container

Measurements were taken in triplicate (*n* = 3) and results expressed as Mean ± SD in centipoise (cP). The viscometer was calibrated using a standard silicone oil reference fluid prior to each measurement session.

#### 4.3.3. Irritancy Test

##### Preliminary Skin Irritancy Test Methodology

A preliminary skin irritancy test was conducted to evaluate the potential of the developed cream formulation to induce cutaneous irritation in albino rats. This study was performed in accordance with ethical guidelines and received approval from the Committee for the Purpose of Control and Supervision of Experiments on Animals (CPCSEA), with ethical approval number 878/ac/05/CPCSEA/23/11.

Rationale for F2 selection: Preliminary antioxidant screening demonstrated F2 provided optimal balance—significant activity (36.1% DPPH scavenging) with excellent physicochemical properties (pH 5.8 ± 0.1, spreadability 11.8 ± 0.3 cm·g/s, viscosity 460,000 ± 26,000 cP). F1 showed marginal activity (6.8%); F3 risked physical instability at higher extract levels.

Test Subjects: The test was conducted on six healthy albino rats; animals were housed under standard laboratory conditions with free access to food and water.

Test Procedure (Patch Test):

1. Preparation: Prior to the test, the dorsal fur of each rat was carefully shaved in two defined areas (e.g., 2 cm × 2 cm) on either side of the spine. This area was selected for its accessibility and ease of observation.
2. Application: A measured amount (0.5 g) of the cream formulation was applied to one of the shaved areas. The application was covered with a non-occlusive patch to ensure contact with the skin while allowing for some air circulation.
3. Control: On the contralateral shaved area of the same rat, a control substance (the cream base without the *Bougainvillea* extract or sterile saline) was applied and covered with a similar non-occlusive patch.
4. Observation Period: The patches were removed after a predetermined period (4 h) to assess initial reactions. The application sites were then observed at specific time intervals: 0.5, 1, 2, 4, 24, 48, and 72 h post-patch removal.
5. Assessment Parameters: The following parameters were assessed visually
  - Erythema: The degree of redness was graded on a scale of 0 (no erythema) to 4 (severe erythema).
  - Edema: The degree of swelling was graded on a scale of 0 (no edema) to 4 (severe edema).
  - Irritation: Any other signs of irritation, such as scaling, dryness, fissuring, or changes in skin texture, were noted and graded based on severity.
  - Inflammation: Any signs of inflammation, such as localized heat or tenderness, were recorded.

#### 4.3.4. Antioxidant Activity Assessment

The antioxidant activity of the *Bougainvillea* extract cold cream (F1–F3) was preliminarily assessed using two complementary methods [4,13,14]:

- Potassium Permanganate Test:

This test, based on the reduction of potassium permanganate (KMnO_4_), was performed as follows:

1. A 0.5% *w*/*v* solution of KMnO_4_ was prepared in distilled water (deep purple color).
2. 0.5 g of the cream formulation (10%, 20%, and 30% *w*/*v*) were separately diluted with 10 mL distilled water.
3. A standard solution of ascorbic acid 10 mg/10 mL = 0.1% *w*/*v* (positive control) was prepared. Distilled water served as the negative control.
4. Four test tubes were labeled: 10% cream, 20% cream, 30% cream, and positive control (ascorbic acid).
5. One drop of the KMnO_4_ solution was added to each test tube.
6. The change in color (from purple to brownish-yellow) was observed and recorded (Table 19). Decolorization of the KMnO_4_ solution indicates the presence of reducing agents of antioxidants [32].

It is acknowledged that the KMnO_4_ reduction test is a non-specific colorimetric screen that detects the presence of reducing agents broadly, without distinguishing between individual antioxidant compounds or providing quantitative data suitable for dose–response analysis. This test was employed exclusively as a rapid preliminary screen during the early formulation development phase to confirm the presence of reducing activity before committing to the more resource-intensive DPPH assay. The results of this test are reported in Table 11 for transparency but are not used to support any quantitative antioxidant conclusions in this study.

b. DPPH Assay:

The DPPH (2,2-diphenyl-1-picrylhydrazyl) assay, a widely used method for assessing free radical scavenging activity [33], was conducted as follows:

Sample preparation: 0.5 g cream (F1, F2, or F3; equivalent to ~0.02 mL at density 0.95 g/mL) dissolved in 10 mL methanol by vortexing/sonication (10 mg/mL stock).

Working solution: 0.2 mL stock + 1.8 mL methanol = final 0.1 mg/mL tested.

i. Extraction of Active Compounds from Cream Matrix

Prior to the DPPH assay, active compounds were extracted from the semisolid cream matrix using a validated solvent extraction procedure. Briefly, 0.5 g of each formulation (F1, F2, and F3) was accurately weighed and dispersed in 10 mL of methanol in a stoppered glass tube. The dispersion was vortex-mixed at high speed for 2 min to disrupt the emulsion structure, followed by ultrasonication at 25 ± 2 °C for 15 min to ensure maximum release of phenolic compounds from the cream base. The mixture was then centrifuged at 3000 rpm for 10 min at room temperature. The clear supernatant was carefully collected, filtered through a 0.45 μm nylon membrane filter, and used directly for the DPPH radical scavenging assay. The blank cream base (without extract) was subjected to the same extraction procedure and used as the matrix control.

ii. Extraction Recovery Validation

To confirm the efficiency and reproducibility of the extraction procedure, a recovery study was performed using the blank cream base spiked with known concentrations of quercetin as a representative marker compound. The blank cream base was spiked at two concentration levels—0.5 mg/g and 1.0 mg/g—and subjected to the extraction procedure described above in triplicate (*n* = 3 per level). The extracted quercetin concentration was quantified by HPTLC densitometry against a pre-validated calibration curve. Mean percentage recoveries of 96.4 ± 1.8% and 97.1 ± 1.3% were obtained at the 0.5 mg/g and 1.0 mg/g spiking levels, respectively, confirming that the extraction procedure achieves quantitative and reproducible recovery of phenolic actives from the cream matrix. These recovery values confirm the reliability of the DPPH data reported for F1, F2, and F3.

iii. DPPH Assay Procedure
  1. A 0.2 mL aliquot of the methanol extract supernatant (diluted to 0.1 mg/mL working concentration) was transferred to a test tube.
  2. 2 mL of DPPH solution was added to the test tube.
  3. The mixture was incubated in the dark for 1 h.
  4. The absorbance of the solution was measured at 517 nm using a UV-Vis spectrophotometer.
  5. The difference in absorbance between the DPPH solution and the DPPH solution with the cream sample was calculated.
  6. Ascorbic acid was used as a standard, and the percentage scavenging activity was calculated using the following formula:

% Scavenged DPPH = [(Abs. of control − Abs. of sample)/Abs. of control] × 100

where:

- Abs. of control = Absorbance of DPPH solution without sample
- Abs. of sample = Absorbance of DPPH solution with cream sample
- Controls:
- Blank: 2 mL DPPH (0.1 mM) + 0.2 mL methanol
- Negative: Negative control: Blank cream base extracted by the same procedure as diluted to 0.1 mg/mL equivalent concentration in methanol
- Positive: Ascorbic acid (0.1 mg/mL)

c. IC_50_ Determination:

Cream samples (F1–F3), extracted, were diluted with methanol to working concentrations of 0.5%, 1.0%, 1.5%, and 2.0% *w*/*v*. Each dilution (0.2 mL) was added to 2 mL DPPH (0.1 mM), incubated for 1 h in the dark, and absorbance measured at 517 nm. % Scavenging was plotted vs. log[concentration] and IC_50_ determined by non-linear regression (GraphPad Prism v8.0, *n* = 3).

#### 4.3.5. Stability Studies

The stability of the prepared cream was evaluated by storing it at controlled conditions of 40 ± 2 °C and 75 ± 5% RH for a period of three months [20]. Samples were withdrawn at 0, 30, 60, and 90 days and examined for any changes in [21,31].

- Physical appearance (color, odor, texture, phase separation)
- Antioxidant activity (using the methods described above) [14].

These stability studies provided information on the shelf life and storage requirements of the formulated cream.

##### HPTLC-Based Marker Compound Stability Assessment

In addition to antioxidant activity monitoring, the chemical stability of the marker compounds quercetin and pinitol was evaluated over the accelerated stability period using HPTLC densitometric quantification. Samples of F2 and F3 were withdrawn at Days 0, 30, 60, and 90 from the accelerated storage conditions (40 ± 2 °C/75 ± 5% RH). At each time point, 1.0 g of each formulation was extracted using the validated methanol extraction procedure described in Section 4.3.4. The clear methanol extract was filtered through a 0.45 μm nylon membrane and spotted onto pre-coated silica gel 60F_254_ HPTLC plates in triplicate (*n* = 3 per time point).

Plates were developed using the mobile phases described in procedure and scanned using a CAMAG TLC Scanner 3 at 366 nm (quercetin) and 520 nm (pinitol, post-derivatization with anisaldehyde-sulfuric acid reagent). The concentration of each marker at each time point was calculated by interpolation from the pre-validated calibration curves. Results were expressed as percentage retention relative to the Day 0 value:

% Marker Retention = (Concentration at time point/Concentration at Day 0) × 100

Statistical comparison between Day 0 and Day 90 values was performed using a paired Student’s *t*-test, with *p* > 0.05 considered indicative of no significant degradation.

#### 4.3.6. Statistical Analysis

All experimental data were obtained in triplicate (*n* = 3) and are expressed as Mean ± Standard Deviation (SD) unless otherwise stated. Statistical analyses were performed using GraphPad Prism v9.0 (GraphPad Software, San Diego, CA, USA), and differences were considered statistically significant at *p* < 0.05. The following statistical methods were applied to each dataset:

(i) One-Way Analysis of Variance (ANOVA) with Tukey’s HSD Post Hoc Test: One-way ANOVA was applied to compare means across all formulation groups (F1, F2, F3, and cream base control) for the following datasets: viscosity measurements (Table 8), DPPH radical scavenging activity (%) (Table 12), IC_50_ values (Table 13), and ex vivo quercetin permeation flux data (Table 10). Where ANOVA indicated a statistically significant difference (*p* < 0.05), Tukey’s Honestly Significant Difference (HSD) post hoc test was applied to identify which specific pairs of formulation groups differed significantly from one another. Results of post hoc comparisons are indicated by distinct superscript letters in the corresponding tables and figures, where groups sharing the same letter are not significantly different.
(ii) Paired Student’s *t*-Test: A paired Student’s *t*-test was applied to compare Day 0 vs. Day 90 values for the following accelerated stability datasets: DPPH radical scavenging activity retention (F2 and F3) and HPTLC-based quercetin and pinitol percentage retention (F2 and F3) (Table 14). This test was selected as the same formulation samples were measured at two time points under matched conditions. A *p*-value > 0.05 was interpreted as indicating no statistically significant degradation over the storage period.
(iii) Linear Regression Analysis: Linear regression was applied to construct HPTLC calibration curves for quercetin and pinitol quantification, covering the concentration range of 0.1–3.0 μg/band. The linearity of each calibration curve was assessed by the correlation coefficient (R^2^), with R^2^ > 0.998 considered acceptable. Linear regression was also applied to the cumulative ex vivo permeation vs. time plots (linear phase: 1–8 h) to calculate the permeation flux (J, μg/cm^2^/h) for each formulation.
(iv) Release Kinetic Modeling: Cumulative ex vivo quercetin permeation data were fitted to zero-order, first-order, Higuchi, and Korsmeyer-Peppas mathematical models using the DD Solver add-in (Microsoft Excel, version 2019). Model selection was based on the highest R^2^ value. The Higuchi model was confirmed as the best fit for all three formulations (R^2^ > 0.99).
(v) IC_50_ Determination: IC_50_ values for DPPH radical scavenging activity were determined by non-linear regression analysis using a sigmoidal dose–response curve fitting model in GraphPad Prism v9.0. Percentage scavenging activity was plotted against the logarithm of concentration (% *w*/*v*), and the IC_50_ was defined as the concentration producing 50% inhibition of DPPH radical activity.

#### 4.3.7. Ex Vivo Skin Permeation Study (Franz Diffusion Cell)

Ex vivo permeation of quercetin from the cold cream formulations (F1, F2, and F3) was evaluated using Franz diffusion cells (diffusion area 3.14 cm^2^, receptor compartment volume 15 mL) with freshly excised goat skin as the permeation barrier. Goat skin was obtained from a local slaughterhouse (Anantapuramu, Andhra Pradesh, India) immediately after sacrifice and used within 2 h of collection. The subcutaneous fat was carefully removed using a scalpel, and the skin was washed with physiological saline (0.9% NaCl). The skin thickness was measured using a digital vernier caliper and confirmed to be uniform across all samples (0.5 ± 0.05 mm). Skin integrity was verified prior to each experiment by measuring transepidermal electrical resistance (TEER); only membranes with TEER > 1 kΩ were used to ensure barrier integrity.

The prepared goat skin was mounted between the donor and receptor compartments with the stratum corneum side facing the donor compartment. The receptor compartment was filled with PBS:ethanol (80:20 *v*/*v*) to maintain sink conditions and stirred continuously at 300 rpm using a magnetic stirrer at 32 ± 0.5 °C to simulate skin surface temperature.

One gram of each formulation (F1, F2, and F3) was applied uniformly to the stratum corneum surface of the mounted skin in the donor compartment and sealed with Parafilm to prevent evaporation. Aliquots of 1.0 mL were withdrawn from the receptor compartment at predetermined time intervals (1, 2, 4, 6, 8, 12, and 24 h) and immediately replaced with an equal volume of fresh receptor medium to maintain sink conditions. The withdrawn samples were filtered through a 0.45 μm nylon membrane filter and analyzed for quercetin content by HPTLC densitometry at 366 nm against a pre-validated calibration curve.

Cumulative quercetin permeation per unit area (μg/cm^2^) was plotted against time (hours). Permeation flux (J, μg/cm^2^/h) was calculated from the slope of the linear portion of the cumulative permeation vs. time curve. Release kinetics were evaluated by fitting the cumulative permeation data to zero-order, first-order, Higuchi, and Korsmeyer–Peppas mathematical models using DD Solver add-in (Microsoft Excel). The best-fit model was selected based on the highest correlation coefficient (R^2^). All experiments were performed in triplicate (*n* = 3).

## 5. Conclusions

This study successfully developed and comprehensively characterized three *Bougainvillea glabra* cold cream formulations (F1: 10%, F2: 20%, F3: 30% *w*/*w* extract) using an integrated approach combining HPTLC-based chemical standardization, validated antioxidant assessment, ex vivo skin permeation evaluation, and accelerated stability profiling. Quantitative HPTLC confirmed quercetin (4.82 ± 0.14 mg/g) and pinitol (2.31 ± 0.09 mg/g) as reproducible chemical markers, enabling reliable batch standardization. The HPTLC analytical method was validated and demonstrating excellent accuracy (mean % recovery: 99.21% for quercetin and 98.60% for pinitol) and precision (% RSD ≤ 1.70% for both intraday and interday measurements), confirming its suitability for routine quantitative estimation of these markers in the developed formulations.

All formulations demonstrated acceptable physicochemical stability and sensory acceptability. F3 emerged as the optimal candidate through multi-criteria analysis, offering the highest antioxidant potency (IC_50_ = 1.3 ± 0.1% *w*/*v*), greatest cumulative quercetin skin permeation (51.3 ± 2.8 μg/cm^2^ at 24 h), and excellent marker compound retention after 90 days of accelerated storage (quercetin 99.1 ± 0.3%, pinitol 98.3 ± 0.4%). Primary dermal irritation testing confirmed a non-irritating preliminary safety profile, and ex vivo tape-strip analysis provided preliminary evidence of antioxidant compound delivery to the stratum corneum.

The present study establishes preliminary dermal safety and in vitro antioxidant efficacy for the *B. glabra* cold cream platform under the conditions tested. These findings suggest that the developed formulation may serve as a promising candidate for further preclinical and clinical investigation. Controlled clinical evaluation in human subjects with oxidative stress-related dermatological conditions, long-term real-time stability assessment under ICH Zone IV conditions, and ex vivo permeation studies using human skin explants represent essential future research directions before definitive therapeutic claims can be substantiated.

## Figures and Tables

**Table 1:** HPTLC R_f_ values and color characteristics of markers in *B. glabra*.

S. No.	Compound/Sample	R_f_ Value	Fluorescence
1	Quercetin (Standard)	0.52	Yellow Fluorescence
2	Pinitol (Standard)	0.35	Brownish-grey
3	*B. glabra* Extract	0.52	Yellow Fluorescence
4	*B. glabra* Extract	0.35	Brownish-grey

**Table 2:** Quantitative marker content showing calibration parameters, limits of detection and quantification, and marker content in the dry extract and formulations (F1–F3).

Marker	Calibration Range (μg/Band)	R^2^	LOD (μg/Band)	LOQ (μg/Band)	Content in Dry Extract (mg/g)	F1 (mg/g)	F2 (mg/g)	F3 (mg/g)
Quercetin	0.1–3.0	0.9985	0.03	0.09	4.82 ± 0.14	0.48 ± 0.01	0.97 ± 0.02	1.45 ± 0.03
Pinitol	0.1–3.0	0.9978	0.04	0.12	2.31 ± 0.09	0.23 ± 0.01	0.46 ± 0.01	0.69 ± 0.02

Note: Values expressed as Mean ± SD (*n* = 3). LOD and LOQ determined by signal-to-noise ratio method (S/N = 3 and 10, respectively).

**Table 3:** Accuracy (Recovery %) of the HPTLC method for quercetin and pinitol.

Marker	Spike Level	Initial Amount (μg/Band)	Amount Added (μg/Band)	Total Amount Found (μg/Band)	% Recovery	Mean % Recovery ± SD
Quercetin	50%	1.0	0.5	1.48	98.67	
	100%	1.0	1.0	1.99	99.50	99.21 ± 0.47
	150%	1.0	1.5	2.49	99.47	
Pinitol	50%	1.0	0.5	1.47	98.00	
	100%	1.0	1.0	1.98	99.00	98.60 ± 0.53
	150%	1.0	1.5	2.47	98.80	

Note: Values expressed as Mean ± SD (*n* = 3). Recovery determined by standard addition method at 50%, 100%, and 150% spike levels. Acceptable recovery range: 98–102%.

**Table 4:** Precision of the HPTLC method for quercetin and pinitol.

Marker	Concentration (μg/Band)	Intraday Precision (*n* = 3)		Interday Precision (*n* = 3)	
		Measured Conc. ± SD	% RSD	Measured Conc. ± SD	% RSD
Quercetin	0.5	0.49 ± 0.004	0.82	0.48 ± 0.006	1.25
	1.5	1.49 ± 0.011	0.74	1.47 ± 0.016	1.09
	2.5	2.48 ± 0.014	0.56	2.46 ± 0.023	0.93
Pinitol	0.5	0.48 ± 0.005	1.04	0.47 ± 0.008	1.70
	1.5	1.48 ± 0.013	0.88	1.46 ± 0.021	1.44
	2.5	2.47 ± 0.019	0.77	2.44 ± 0.031	1.27

Note: Values expressed as Mean ± SD (*n* = 3). %RSD = percentage relative standard deviation. Intraday precision: three replicate measurements on the same day. Interday precision: measurements on three consecutive days. Acceptance criterion: %RSD < 2%.

**Table 5:** Organoleptic properties of cream.

S. No.	Property	Result
1	Colour	Green
2	Odor	Characteristic
3	Appearance	Semi-solid

**Table 6:** Preliminary phytochemical screening results.

Test	Observation	Result
Flavonoids	Purple to violet color	+
Phenolics	Blue-black to dark green	+
Alkaloids	Orange/cream/brown ppt	+++
Tannins	Blue-black	+
Saponins	Stable froth (≥15 min)	+
Terpenoids	Red-brown ring	+

Note: + = present; +++ = strongly present. Tests performed in triplicate.

**Table 7:** pH of the formulated cream.

S. No	Formulation	pH
1	F1	5.9 ± 0.1
2	F2	5.8 ± 0.1
3	F3	5.7 ± 0.1
4	Cream base	5.9 ± 0.1

**Table 8:** Spreadability of the formulation.

Trial	Time (s)	Spreadability (cm·g/s)
1	8	12.5 cm·g/s
2	9	11.11 cm·g/s
3	8.5	11.76 cm·g/s
Mean ± SD	8.5 ± 0.5	11.79 ± 0.70 cm·g/s

**Table 9:** Rheological characterization.

Reading	Viscosity (cP)
Cream Base	380,000 ± 15,000 cP
F1 (10%)	440,000 ± 22,000 cP
F2 (20%)	460,000 ± 26,000 cP
F3 (30%)	455,000 ± 20,000 cP

**Table 10:** Viscosity ANOVA result.

Comparison	*p*-Value	Significance
Base vs. F1	0.003	*p* < 0.01
Base vs. F2	0.001	*p* < 0.01
Base vs. F3	0.002	*p* < 0.01
F1 vs. F2	0.78	ns
F2 vs. F3	0.89	ns
F1 vs. F3	0.92	ns

ANOVA: F = 12.34, *p* = 0.002 (significant), ns = Not significant.

**Table 11:** Ex vivo cumulative quercetin permeation data (Franz Diffusion Cell, Goat Skin Membrane, 32 ± 0.5 °C, *n* = 3).

Time (h)	F1 (μg/cm^2^)	F2 (μg/cm^2^)	F3 (μg/cm^2^)
1	4.5 ± 0.3	7.1 ± 0.5	9.5 ± 0.6
2	7.8 ± 0.5	13.4 ± 0.8	17.1 ± 1.1
4	11.4 ± 0.8	19.8 ± 1.2	26.3 ± 1.6
6	13.5 ± 1.0	24.9 ± 1.5	36.2 ± 2.0
8	15.7 ± 1.1	28.1 ± 1.8	45.8 ± 2.3
12	17.5 ± 1.2	32.0 ± 2.0	48.1 ± 2.5
24	18.2 ± 1.4	34.7 ± 2.1	51.3 ± 2.8

Cumulative quercetin permeation expressed as Mean ± SD. Permeation area = 3.14 cm^2^. Receptor medium: PBS:ethanol (80:20 *v*/*v*), pH 7.4.

**Table 12:** Skin Irritation Test. The erythema and edema scores are represented as “Erythema/Edema.” (0 = none, 1 = slight, 2 = moderate, 3 = severe).

Rat ID	Treatment	1 h	4 h	24 h	48 h	72 h	Overall Assessment
1	Bougainvillea Cream	0/0	0/0	0/0	0/0	0/0	No Irritation
2		0/0	0/0	0/0	0/0	0/0	No Irritation
3		0/0	0/0	0/0	0/0	0/0	No Irritation
4		0/0	0/0	0/0	0/0	0/0	No Irritation
5		0/0	0/0	0/0	0/0	0/0	No Irritation
6 (control)		0/0	0/0	0/0	0/0	0/0	No Irritation

Statistical analysis: One-way ANOVA with Tukey’s HSD post hoc test applied to flux values. Data expressed as Mean ± SD (*n* = 3).

**Table 13:** Qualitative screening data—potassium permanganate reduction test for *Bougainvillea* cold cream formulations.

Test Variable	Trial 1	Trial 2	Interpretation
A. 10% Formulated Cream (F1)	Light purple (lighter than original)	Light purple (lighter than original)	Negative (but some color change)
B. 20% Formulated Cream (F2)	Brownish-yellow	Brownish-yellow	Positive for antioxidant activity (decolorization)
C. 30% Formulated Cream (F3)	Brownish-yellow	Light yellow	Positive for antioxidant activity (decolorization)
D. Positive Control (Ascorbic Acid)	Yellow	Yellow	Positive for antioxidant activity (decolorization)

Note: These results represent preliminary qualitative observations only and are not used to support quantitative antioxidant conclusions. All quantitative antioxidant data are derived from the validated DPPH assay (Table 14).

**Table 14:** Antioxidant activity of *Bougainvillea* cream (DPPH assay).

Sample	Mean Absorbance ± SD (517 nm)	DPPH Radical Scavenging Activity (%)	Statistical Significance
DPPH solution (Blank)	3.426 ± 0.226	--	
Blank Cream Base (Control)	3.193 ± 0.157	0.0 ± 0.01%	—
Ascorbic Acid (Standard)	0.705 ± 0.052	79.422 ± 0.1%	a
10% Formulated Cream	3.193 ± 0.157	6.801 ± 0.2%	b
20% Formulated Cream	2.188 ± 0.509	36.135 ± 0.5%	c
30% Formulated Cream	1.484 ± 0.105	56.683 ± 1.1%	d

Statistical analysis: One-way ANOVA, F = 245.6, *p* < 0.0001; Tukey’s HSD post hoc test. Different superscript letters indicate significant difference (*p* < 0.05). Note: % Scavenging = [(Abs_blank − Abs_sample)/Abs_blank] × 100, Different superscript letters = significant difference (Tukey’s test, *p* < 0.05). Blank = DPPH solution + methanol (no sample).

**Table 15:** IC_50_ analysis.

Sample	IC_50_ (% *w*/*v*)	95% CI	Statistical Grouping
Cream Base	>5.0	0	0
F1 (10%)	4.2 ± 0.4	3.4–5.0	b
F2 (20%)	2.1 ± 0.3	1.7–2.5	b
F3 (30%)	1.3 ± 0.1	1.1–1.5	a
Ascorbic Acid	0.08 ± 0.01	0.06–0.10	a

*n* = 3, Statistical analysis: One-way ANOVA, F = 45.2, *p* < 0.0001; IC_50_ determined by non-linear regression (GraphPad Prism v9.0). Different letters indicate significant difference (Tukey’s HSD, *p* < 0.05).

**Table 16:** Accelerated Stability Study—DPPH antioxidant activity and HPTLC marker compound retention (40 ± 2 °C/75 ± 5% RH, *n* = 3).

Time (Days)	F2 DPPH Scavenging (%)	F3 DPPH Scavenging (%)	F2 Quercetin Retention (%)	F3 Quercetin Retention (%)	F2 Pinitol Retention (%)	F3 Pinitol Retention (%)
0	36.135 ± 0.51	56.683 ± 1.11	100.0	100.0	100.0	100.0
30	35.753 ± 0.40	56.449 ± 1.00	99.4 ± 0.3	99.6 ± 0.2	99.1 ± 0.4	99.3 ± 0.3
60	35.928 ± 0.50	56.537 ± 0.90	99.0 ± 0.4	99.4 ± 0.3	98.5 ± 0.4	98.8 ± 0.3
90	35.509 ± 0.45	56.126 ± 0.95	98.6 ± 0.4	99.1 ± 0.3	97.9 ± 0.5	98.3 ± 0.4
*p*-value (Day 0 vs. 90)	ns	ns	ns	ns	ns	ns

Statistical analysis: Paired Student’s *t*-test (Day 0 vs. Day 90). ns = not significant (*p* > 0.05, *n* = 3). DPPH values expressed as Mean ± SD. Marker retention expressed as % of Day 0 concentration (Mean ± SD). ns = not significant (paired *t*-test, *p* > 0.05). Activity retention: F2 DPPH = 98.3%; F3 DPPH = 99.0% after 90 days.

**Table 17:** Optimized formulation based on multiple factors.

Parameter	F1 (10%)	F2 (20%)	F3 (30%)	Winner
IC_50_ (Potency)	4.2 ± 0.4%	2.1 ± 0.2%	1.3 ± 0.1%	F3
Viscosity	440k cP	460k ± 26k	455k cP	F2
Spreadability	Good	11.8 ± 0.3	Good	F2
pH	5.9	5.8 ± 0.1	5.7	F3
Stability (90d)	Stable	Stable	Stable	All
Safety	Non-irritant	Non-irritant	Non-irritant	All

**Table 18:** Composition of *B. glabra* cold cream formulations.

Ingredient	INCI Name	Function	F1 (10%)	F2 (20%)	F3 (30%)
*B. glabra* Extract	*Bougainvillea glabra* Leaf Extract	Antioxidant	10 g	20 g	30 g
Beeswax	Cera Alba	Stiffening agent	8.0 g	8.0 g	8.0 g
Liquid Paraffin	Paraffinum Liquidum	Emollient	12.5 mL	12.5 mL	12.5 mL
Borax	Sodium Borate	Emulsifier	1.0 g	1.0 g	1.0 g
Methylparaben	Methylparaben	Preservative	0.4 g	0.4 g	0.4 g
Glycerin	Glycerin	Humectant	5 mL	5 mL	5 mL
Perfume (Rose oil)	Fragrance	Fragrance	q.s.	q.s.	q.s.
Purified Water	Aqua	Vehicle	q.s. to 100 g	q.s. to 100 g	q.s. to 100 g

Note: F1–F3: Cold cream formulations with varying extract concentrations (*w*/*w*). q.s. = quantity sufficient.

**Table 19:** KMnO_4_ assay concentrations.

Sample	Mass Taken	Final Volume	Final Conc. (% *w*/*v*)
F1–F3 creams	0.5 g	10 mL	0.5
Cream base	0.5 g	10 mL	0.5
Ascorbic acid	10 mg	10 mL	0.1

## Data Availability

The original contributions presented in this study are included in the article. Further inquiries can be directed to the corresponding author.
